# Predicting who fails to meet the physical activity guideline in pregnancy: a prospective study of objectively recorded physical activity in a population-based multi-ethnic cohort

**DOI:** 10.1186/s12884-016-0985-x

**Published:** 2016-07-26

**Authors:** Kåre Rønn Richardsen, Ragnhild Sørum Falk, Anne Karen Jenum, Kjersti Mørkrid, Egil Wilhelm Martinsen, Yngvar Ommundsen, Sveinung Berntsen

**Affiliations:** 1 Norwegian National Advisory Unit on Women’s Health, Oslo University Hospital, Oslo, Norway; 2 Institute of Health and Society, Department of General Practice, Faculty of Medicine, University of Oslo, Oslo, Norway; 3 Faculty of Health Sciences, Oslo and Akershus University College of Applied Sciences, Oslo, Norway; 4 Oslo Centre for Biostatistics and Epidemiology, Oslo University Hospital, Oslo, Norway; 5 Department of International Public Health, Norwegian Institute of Public Health, Oslo, Norway; 6 Clinic Mental Health and Addiction, Oslo University Hospital, Oslo, Norway; 7 Institute of Clinical Medicine, University of Oslo, Oslo, Norway; 8 Department of Coaching and Psychology, Norwegian School of Sport Sciences, Oslo, Norway; 9 Faculty of Health and Sport Sciences, University of Agder, Kristiansand, Norway

**Keywords:** Physical activity, Pregnancy, Multi-ethnic, Prediction

## Abstract

**Background:**

A low physical activity (PA) level in pregnancy is associated with several adverse health outcomes. Early identification of pregnant women at risk of physical inactivity could inform strategies to promote PA, but no studies so far have presented attempts to develop prognostic models for low PA in pregnancy. Based on moderate-to-vigorous intensity PA (MVPA) objectively recorded in mid/late pregnancy, our objectives were to describe MVPA levels and compliance with the PA guideline (≥150 MVPA minutes/week), and to develop a prognostic model for non-compliance with the PA guideline.

**Methods:**

From a multi-ethnic population-based cohort, we analysed data from 555 women with MVPA recorded in gestational week (GW) 28 with the monitor SenseWear™ Pro3 Armband. Predictor variables were collected in early pregnancy (GW 15). We organized the predictors within the domains health, culture, socioeconomic position, pregnancy, lifestyle, psychosocial factors, perceived preventive effect of PA and physical neighbourhood. The development of the prognostic model followed several steps, including univariate and multiple logistic regression analyses.

**Results:**

Overall, 25 % complied with the PA guideline, but the proportion was lower in South Asians (14 %) and Middle Easterners (16 %) compared with Westerners (35 %). Among South Asians and Middle Easterners, 35 and 28 %, respectively, did not accumulate any MVPA minutes/week compared with 18 % among Westerners. The predictors retained in the prognostic model for PA guideline non-compliance were ethnic minority background, multiparity, high body fat percentage, and perception of few physically active friends. The prognostic model provided fair discrimination between women who did vs. did not comply with the PA guideline.

**Conclusion:**

Overall, the proportion who complied with the PA guideline in GW 28 was low, and women with ethnic minority background, multiparity, high body fat percentage and few physically active friends had increased probability of non-compliance. The prognostic model showed fair performance in discriminating between women who did comply and those who did not comply with the PA guideline.

**Electronic supplementary material:**

The online version of this article (doi:10.1186/s12884-016-0985-x) contains supplementary material, which is available to authorized users.

## Background

Meeting the recommended levels of physical activity (PA) has particular public health importance during pregnancy as both mother and offspring may benefit. Intervention studies have shown that PA reduces the risk of gestational diabetes (GDM) and neonates being large for gestational age [1–4]. Furthermore, GDM predisposes the mother and her offspring for developing type 2 diabetes and obesity in the future [5–7]. While there is a considerable uncertainty around the cost-effectiveness of interventions including PA promotion during pregnancy [8], the potential for health care workers to reach women across social groups is evident. By capitalizing on this window of opportunity, PA promotion during pregnancy may have long-lasting impact on health outcomes and social health inequalities.

For the general population, there is evidence of substantial health benefit from performing 150 min/week of moderate-to-vigorous intensity PA (MVPA) [9–11], and the same activity target is recommended for healthy pregnant women [12,13]. Despite the health-enhancing effects, the proportion of pregnant women who meet the recommended PA levels ranges from 4 to 60 % [14–17]. In addition to true population differences, this partly reflects different guidelines and methods of PA measurement.

Estimates of PA levels in most studies are based on self-reports [18]. Besides the cohort of this study, we are aware of only one other population-based study of PA correlates that includes objectively recorded PA [16]. We have previously reported on objectively recorded MVPA in early pregnancy from the STORK Groruddalen cohort from which we report in the present study [19]. The scarcity of studies based on objective methods means that estimates of PA levels and PA correlates are prone to reporting bias and inaccuracy [20]. Studies based on objective methods are required to contribute new knowledge about groups and individuals at risk of insufficient MVPA at different stages of pregnancy.

Successful promotion of PA in pregnancy depends on interventions that positively modifies PA behaviour and methods to identify individuals and groups at increased risk of not meeting the recommended levels of PA. Prognostic models are tools that combine multiple predictors to obtain an estimate of probability of a future outcome [21]. Prognostic models are distinctively different from etiological models underpinned by causal theory [22], and they may even be non-causal [23]. While prognostic models are more commonly applied to predict disease outcomes, they may also predict lifestyle outcomes [21]. However, there are few examples of prognostic models developed to predict PA [24], and to our knowledge, no previous studies have presented a prognostic model for insufficient MVPA in pregnancy. To make prognostic models relevant for the clinical setting, it is recommended that predictors should originate from low-cost data collection methods that are not burdensome for the patients [25]. At the same time, potential predictors must reflect current evidence on PA correlates. A consistent association has been shown between low PA levels and non-Western ethnicity, low educational level, past pregnancies and low levels of pre-pregnancy PA [16, 19, 26]. Findings are equivocal with respect to the association with maternal age, occupational group, marital status, and smoking [26].

To inform strategies to promote PA among pregnant women in multi-ethnic populations, there is a need for research based on objective measures to obtain valid estimates of PA levels and their distribution in populations. Objectively recorded PA can also enhance prognostic studies to determine insufficient PA, as accurate predictions rely on unbiased PA data. Based on objectively recorded MVPA, our objectives were to describe MVPA levels and compliance with the PA guideline (i.e. ≥150 MVPA minutes/week) in gestational week (GW) 28, and to develop and validate a prognostic model of guideline non-compliance based on clinical data collected in early pregnancy (GW 15).

## Methods

### Population, setting and data collection

Data originated from the population-based STORK Groruddalen cohort study (STORK-G), in which participants were pregnant women from multi-ethnic districts in Oslo [27]. Recruitment took place between May 2008 and May 2010 at three public Child Health Clinics where women received antenatal care. Inclusion criteria were planned birth at either of two study hospitals, ≤20 weeks’ gestation, ability to communicate in Norwegian (or Arabic, English, Sorani, Somali, Tamil, Turkish, Urdu, Vietnamese), and ability to give written consent. Exclusion criteria were pre-gestational diabetes or other conditions necessitating intensive hospital follow-up during pregnancy. In total, 823 women were included at the baseline visit (mean GW 15.1, SD 3.7), while 772 attended the follow-up visit (mean GW 28.3, SD1.3) [27]. Anthropometric measurements were recorded and questionnaire data collected during face-to-face interviews at the baseline visit. If required, the interviewing midwives used translated versions of the questionnaires (in one of the eight languages listed under the inclusion criteria), and professional interpreters assisted during interviews if needed. MVPA was objectively recorded for 4 to 7 days immediately after the follow-up visit. Participants gave informed written consent before participation. The Regional Committee for Medical and Health Research Ethics for South Eastern Norway and The Norwegian Data Inspectorate approved the study protocol. The study methods are described in detail elsewhere [27].

### Primary outcomes

The two primary outcomes were MVPA minutes/week and PA guideline compliance (150 MVPA minutes/week: yes/no). We calculated MVPA minutes/week by multiplying mean MVPA minutes/day by seven (days). MVPA was objectively recorded with the multi-sensor SenseWear™ Pro3 Armband (SWA) (BodyMedia Inc., Pittsburgh, Pennsylvania, USA). The device collects data on acceleration, skin temperature, heat flux and galvanic skin response, while machine learning algorithms produce estimates of energy expenditure based on the incoming data [28]. The SWA provides valid estimates of energy expenditure during pregnancy [29, 30]. The SWA was affixed across the right triceps brachii of the participant at the follow-up visit (GW 28), and she was asked to wear it continuously for the next 4 to 7 days, except during shower/water activities. We downloaded data with the software from the manufacturer (SenseWear™ Professional Research Software Version 6.1, BodyMedia Inc). The summed value of 1-min epochs was used to estimate metabolic equivalents (METs) (1 MET = 3.5 ml O_2_ · kg^−1^ · min^−1^). MVPA was restricted to bouts ≥10 subsequent minute epochs ≥3METs, and these minutes were extracted with SQL Server Management Studio (Microsoft^®^) and SQL Server Express version 11.0.5058.0 (Microsoft^®^). A day of recording was valid if the participant wore the SWA for at least 19.2 h, i.e. 80 % of a 24-h sampling period [31]. In the analysis, we included only data from women with ≥2 valid days of SWA wear time.

### Predictors

We selected candidate predictors for PA guideline non-compliance from data collected by trained midwives at the baseline visit. Ethnicity referred to the participant’s country of birth or the country of birth of the mother of the participant if the mother was born outside Europe or North America. Ethnic categories analysed were Western, South Asian, Middle Eastern and other ethnicity. Occupation was recorded according to the International Standard Classification of Occupations [32]. Occupational groups analysed were managers/° occupations, clerical/care occupations, and elementary occupations/homemakers. Parity was categorised as nullipara, unipara and multipara (≥2 births). Pre-pregnancy PA was self-reported and referred to duration and frequency of pre-defined endurance activities three months pre-pregnancy (running/jogging, bicycling, aerobic classes, dancing, ball sports, swimming and brisk walking/skiing) [33]. We calculated total minutes/week by multiplying minutes/sessions by sessions/week (never, 0.5x/week, 1x/week, 2x/week, 4.5x/week and daily), and the total was dichotomised (150 min/week yes/no). Perception of physically active friends was a measure of the underlying construct descriptive norm, i.e. the participants’ perceptions about the physical activity behaviour in other relevant groups [34]. Friends, and in particular same-aged and female friends, were considered to be significant context-specific groups for physical activity among pregnant women [34]. Hence, building upon the combined friends and family scale developed by Okun and colleagues [35], we modified the scale to include three-items pertaining to perceptions of how many friends, same-aged friends and same-aged female friends who were physically active ≥3x/week. Each item was scored on a 5-point Likert Scale (0 = none, 5 = all). The item loadings derived by exploratory factor analysis ranged from 0.88 to 0.93 while the Cronbach Alpha score was 0.89, indicating a one-factor structure with a high level of internal consistency. The sum score of the three items was median-dichotomized into many versus few physically active friends. Perceived preventive effect of PA was expressed as the sum of scores of nine items (cardiovascular, musculoskeletal, type 2 diabetes, cancer, hypertension, mental illness, overweight/obesity, abdominal/intestinal disease, and, asthma/allergies) scored on 3-point scales (0 = no effect, 1 = little effect, 2 = large effect). Body fat percentage was measured with bioelectric impedance analysis using Tanita-Weight BC-418 MA (Tanita Corp., Tokyo, Japan) [27, 36]. Descriptions of candidate predictors not included in the full model are available as supplementary material [Additional file 1].

### Reasons for missing data

Of the 823 subjects included at baseline, 51 did not attend the follow-up visit in GW 28 due to abortions/preterm birth (*n* = 18) or unknown reasons (*n* = 33). Among the remaining 772 who attended the follow-up visit, reasons for missing MVPA data were: no available SWA due to logistical problems (*n* = 47), the participant declined or was unable to wear the SWA (*n* = 48), or the participant wore the SWA but had insufficient wear time (*n* = 122).

### Statistical analyses

Descriptive characteristics are presented as mean, median, standard deviation (SD), interquartile range and proportions. Group differences are analysed by t-tests and Chi-square tests, as appropriate.

### Development of prognostic model

Development and validation of the prognostic model are reported in accordance with the TRIPOD-statement [21]. To develop the prognostic model, we initially identified potential predictors based on a review of the literature. The predictors were organized into eight domains (*health, culture, socioeconomic position, pregnancy, lifestyle, psychosocial factors, perceived preventive effect of PA and physical neighbourhood*). Following removal of predictors with *p* > 0.2 in univariate regression [37], candidate predictors in seven of the domains remained (no predictors remained in the domain *physical neighbourhood*). To enhance the prediction, we included the strongest predictor from each of the seven domains in the full model [24]. Starting with the full model, we performed multiple logistic regression analysis with backward elimination to determine the final prognostic model. Further details are presented as supplementary material [Additional file 2].

Calibration of the final model was assessed by the Hosmer-Lemeshow test. A calibration plot presents the test result graphically by showing agreement between observed and predicted values by sample deciles, where perfect predictions align along the 45° line [25]. We assessed the ability of the model to discriminate between women who complied vs. did not comply with the PA guideline by the Area Under the Receiver Operating Characteristic (AU-ROC) curve [25].

### Internal validation of prognostic model

We performed a bootstrap resampling procedure using 1,000 iterations to correct for overfitting [38, 39]. The shrunk model consisted of corrected coefficients calculated as the average of the coefficients from the 1,000 bootstrap samples. As internal validation of the discrimination, we calculated the bias-corrected AU-ROC (i.e. the average of all 1,000 AU-ROCs) with bootstrap generated 95 % CI.

*P*-values ≤0.05 were considered statistically significant. All analyses were performed in Stata 13 [40].

### Sensitivity analysis

We analysed sensitivity to number of SWA days by repeating the multiple logistic regression with backward elimination, starting with the full mode, using observations with ≥4 valid SWA days and comparing the resultant odds ratios with the odds ratios from the original model based on observations with ≥2 valid SWA days.

Starting with the full model, we performed multiple logistic regression analysis with backward elimination.

## Results

### Sample characteristics

The sample consisted of 555 participants with valid SWA data. At the baseline visit, mean (min-max/SD) age was 30.1 years (19.3–45.1/4.9) and pre-pregnancy body mass index (BMI) was 24.4 kg/m^2^ (14.9–49.2/4.8), while body fat percentage was 33.1 % (10.9–53.5/7.4) (Table 1). SWA wear time mean (SD) was 3.6 (1.0) days. Compared with women not eligible for the analyses, the sample was marginally older, had marginally lower body mass index, and had a higher proportion of Western women (Table 1).

**Table 1:** Characteristics of cohort at stratified by eligibility for analysis

	Valid SWA data (eligible)		Without valid SWA data (not eligible)		
	(*n* = 555)		(*n* = 268)		
	*n*	%	*n*	%	*P*-value ^a^
Ethnicity					<0.01
South Asian	125	(22.5)	75	(28.0)	
Middle Eastern	75	(13.5)	51	(19.0)	
Other ethnicity	100	(18.0)	61	(22.8)	
Western	255	(46.0)	81	(30.2)	
Occupation					0.20
Elementary occupations and homemakers	150	(27.4)	86	(33.5)	
Clerical/care occupations	196	(35.8)	86	(33.5)	
Manager/° occupations	202	(36.8)	85	(33.0)	
Missing	7		11		
Education					0.39
<10 years	85	(15.4)	48	(18.2)	
10–12 years	216	(39.0)	108	(40.9)	
University or college	252	(45.6)	108	(40.9)	
Missing	2		4		
Parity					0.11
None (nulliparous)	253	(45.6)	128	(47.8)	
1 (uniparous)	201	(36.2)	79	(29.5)	
≥2 (multiparous)	101	(18.2)	61	(22.7)	
Smoking 3 months pre-pregnancy					0.96
Non-smoker	455	(82.4)	218	(82.6)	
Irregular or daily smoker	97	(17.6)	46	(17.4)	
Missing	3		4		
Self-reported pre-pregnancy PA					0.16
≥150 min/wk	220	(40.6)	91	(35.4)	
<150 min/wk	322	(59.4)	166	(64.6)	
Missing	13		11		
Health pre-pregnancy					0.78
Poor/not too good	58	(10.6)	30	(11.5)	
Good	279	(50.7)	137	(52.3)	
Very good	213	(38.7)	95	(36.2)	
Missing	5		6		
Pelvic girdle-/lumbopelvic pain					0.87
Yes	228	(41.8)	107	(41.2)	
No	318	(58.2)	153	(58.8)	
Missing	9		8		
	Mean	SD	Mean	SD	*P*-value ^b^
Age (years)	30.1	(4.9)	29.3	(4.8)	0.02
BMI pre-pregnancy	24.4	(4.8)	25.0	(4.9)	<0.01
Body fat percentage	33.1	(7.4)	34.5	(7.3)	0.15

*SD* standard deviation, *SWA* Sensewear Armband, *BMI* body mass index

^a^ Chi-Square test

^b^ *t*-test

### PA guideline compliance (unadjusted analyses)

Overall, 25 % complied with the PA guideline in GW 28. By ethnic groups, 35 % of Westerners complied with the guideline, 14 % of South Asians and 16 % of Middle Easterners. (Table 2). Having university/college education, manager/° occupations, being nullipara, and having a low body fat percentage were all associated with compliance (Table 2).

**Table 2:** Moderate-to-vigorous intensity physical activity and compliance with the physical activity guideline at follow-up visit (*n* = 555)

	MVPA min/wk ^a^		PA guideline compliance ^a^		
	Median	IQR	*n*	%	*P*-value ^b^
Overall	64.4	(12.8–152.3)	141	(25.4)	
Ethnicity					<0.01
South Asian	23.8	(0–119.0)	18	(14.4)	
Middle Eastern	35.0	(0–95.7)	12	(16.0)	
Other ethnicity	59.3	(0–137.1)	22	(22.0)	
Western	84.0	(26.8–183.4)	89	(34.9)	
Occupation					<0.01
Elementary occupations & homemakers	26.8	(0–99.8)	23	(15.3)	
Clerical/care occupations	55.4	(0–125.1)	41	(20.9)	
Manager/° occupations	103.3	(35.0–184.3)	76	(37.6)	
Education					<0.01
<10 years education	47.8	(0–117.2)	15	(17.7)	
10–12 years education	38.5	(0–107.6)	42	(19.4)	
University/college	84.0	(23.3–177.0)	84	(33.3)	
Parity					<0.01
None (nulliparous)	78.4	(12.8–169.8)	80	(31.6)	
1 (uniparous)	66.5	(21.0–161.0)	54	(26.9)	
≥2 (multiparous)	30.3	(0–85.8)	7	(6.9)	
Planned pregnancy					0.04
Yes	75.6	(19.3–159.3)	114	(27.6)	
No	38.5	(0–108.5)	26	(19.0)	
Smoking 3 months pre-pregnancy					0.50
Non-smoker	65.3	(0–155.8)	118	(25.9)	
Irregular or daily smoker	57.8	(19.3–141.4)	22	(22.7)	
Self-reported pre-pregnancy PA					0.02
≥150 min/week	84.0	(9.6–178.5)	68	(30.9)	
<150 min/week	49.0	(12.8–133.0)	70	(21.7)	
Physically active friends					<0.01
Many	77.0	(19.3–179.7)	81	(31.3)	
Few	51.3	(0–119.0)	60	(20.3)	
Health pre-pregnancy					<0.01
Poor/not too good	40.3	(0–105.0)	8	(13.8)	
Good	51.3	(0–127.8)	60	(21.5)	
Very good	84.0	(23.3–175.0)	72	(33.8)	
Pelvic girdle-/lumbopelvic pain					0.07
Yes	51.9	(6.4–120.8)	48	(21.1)	
No	72.6	(17.5–164.5)	89	(28.0)	
			Mean	SD	*P*-value ^c^
Age, years			29.8	(4.6)	0.39
Body fat percentage			29.7	(7.3)	<0.01

*IQR* interquartile range, *PA* physical activity, *MVPA* moderate-to-vigorous physical activity

^a^ Recorded by Sensewear Armband Pro3

^b^ Chi-square test of difference between categories in PA guideline compliance

^c^ Unpaired *t*-test

### MVPA minutes/week (unadjusted analyses)

Overall, 25 % of the sample recorded no MVPA minutes/week in bouts ≥10 min. The proportion was 18 % for Westerners, 35 % for South Asians and 18 % for Middle Easterners. Differences in MVPA minutes/week were observed across ethnic groups, educational categories, parity categories and pre-pregnancy PA (Table 2).

### Prognostic model

After elimination of predictors from the original list of candidate predictors [Additional file 2], remaining predictors included in the full model were ethnicity (*P* < 0.01), occupation (*P* < 0.01), parity (*P* < 0.01), pre-pregnancy PA (*P* = 0.02), physically active friends (*P* < 0.01), perceived preventive effect of PA (*P* = 0.14) and body fat percentage (*P* < 0.01). After multiple logistic regression with backward elimination, the four predictors retained in the final prognostic model were ethnicity, parity, physically active friends and body fat percentage (Nagelkerke R^2^ = 0.14) (Table 3). The sensitivity analysis based on data from participants with ≥4 valid SWA days supported the results in the original prognostic model.

**Table 3:** Odds ratios for not meeting the physical activity guideline by multiple logistic regression analyses (*n* = 535)

Predictors	Final model			Bootstrap validation		
	OR	(95 % CI)	*P*-value	OR	(95 % CI)	*P*-value
Ethnicity (ref: Western)						
South Asian	2.7	(1.5, 4.8)	<0.01	2.7	(1.4, 5.0)	<0.01
Middle Eastern	2.2	(1.1, 4.5)	0.03	2.2	(1.1, 4.5)	0.04
Other	1.8	(1.0, 3.3)	0.05	1.8	(1.0, 3.4)	0.05
Parity (ref: nulliparous)						
1 (uniparous)	1.2	(0.8, 1.8)	0.48	1.2	(0.7, 1.9)	0.50
≥2 (multiparous)	5.3	(2.1, 12.9)	<0.01	5.3	(1.9, 1.6)	<0.01
Physically active friends (ref: many) ^a^						
Few	1.7	(1.1, 2.6)	0.01	1.7	(1.1, 2.7)	0.02
Body fat percentage	1.1	(1.06, 1.13)	<0.01	1.1	(1.06, 1.13)	<0.01
*Constant*	*0.07*	*(0.02 0.21)*	*<0.01*	*0.07*	*(0.02, 0.22)*	*<0.01*

*OR* odds ratio, *CI* confidence interval, *PA* physical activity

^a^Missing values on 20 women

**Fig. 1:**
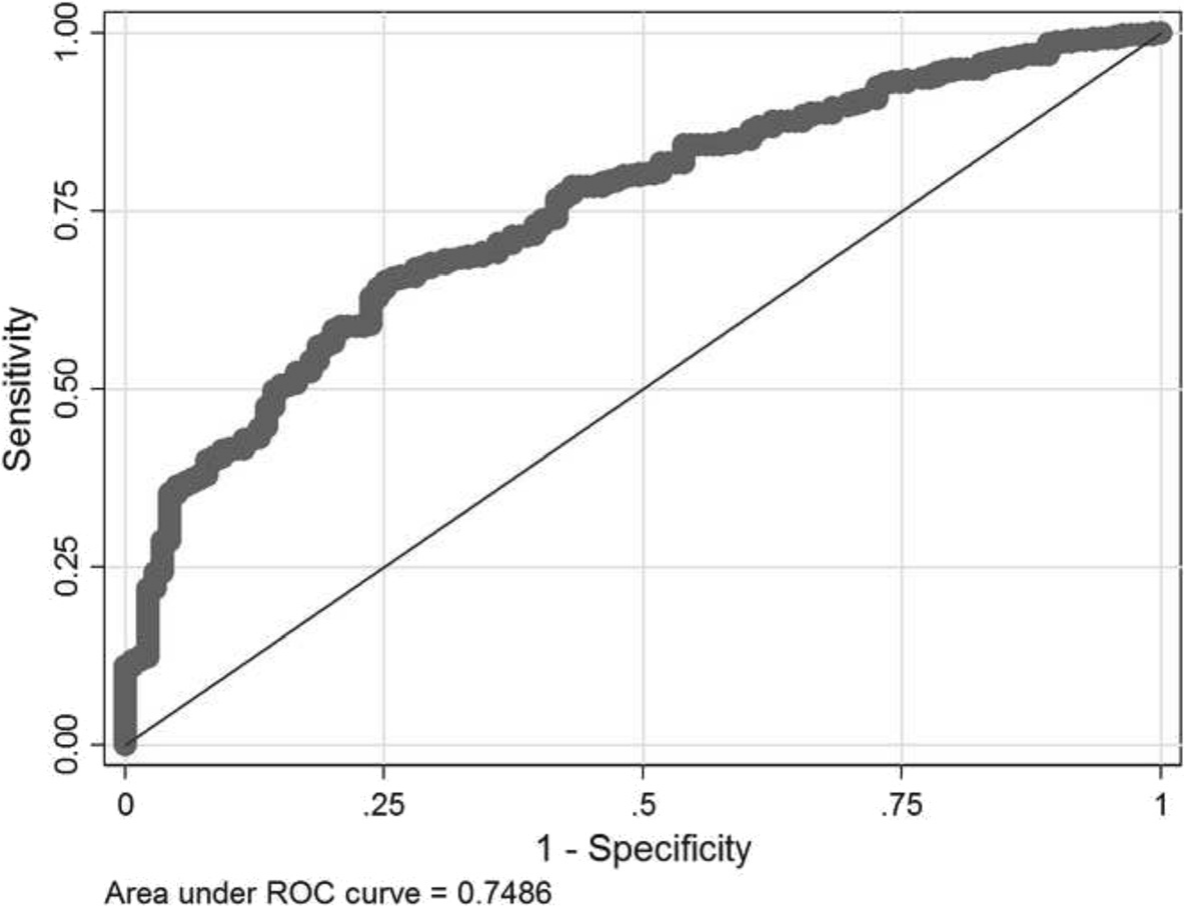
Receiver Operating Characteristics (ROC) curve. The discriminatory power of the prognostic model, expressed as the area under the Receiver Operating Characteristics (ROC) curve

**Fig. 2:**
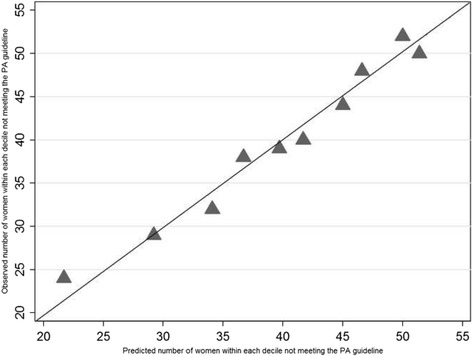
Calibration plot. Triangles (▲) express the agreement between observed and predicted non-compliance with the physical activity guideline for each sample decile. The 45° line represents perfect predictions

The final prognostic model demonstrated fair discrimination between women who complied and did not comply with the PA guideline (AU-ROC = 0.749) (Fig. 1) [41]. The calibration plot (Fig. 2) and the Hosmer-Lemeshow test (*P* = 0.85) based on the final model demonstrated a good match between the predicted and observed outcomes across deciles of the data.

### Model validation

The adjusted coefficients derived by the bootstrap resampling corresponded with the coefficients in the final prognostic model, indicating the model was not overfitted. The bias corrected AU-ROC (95 % CI) was 0.757 (0.638, 0.784), which indicates bias was marginal (−0.008).

An example of risk estimation for sub-groups using the prognostic model shows that the predicted probability of PA guideline non-compliance is 98 % for multiparous South Asian women with few physically active friends and 38 % body fat.

## Discussion

To our knowledge, the STORK-G is the only population-based pregnancy cohort in Europe that includes objectively recorded MVPA. Special efforts were made to recruit ethnic minority women who constitute a growing proportion of pregnant women in Europe. Furthermore, the present study is the first to develop and validate a prognostic model for non-compliance with a PA guideline for pregnant women.

Only 25 % of pregnant women complied with the PA guideline in GW 28. Even more alarming, only 14 % of South Asians and 16 % of Middle Easterners complied, while the prevalence was 35 % among Western women. The prevalence of PA guideline compliance was 33 % among women with university/college education and 19 % among those with <12 years education. One in four never recorded MVPA of at least 10 min duration. The prognostic model showed that ethnic minority background, multiparity, high body fat percentage and few physically active friends predicted non-compliance with the PA guideline. The predicted outcome was correct for three out of four women, which is considered as a fair discriminatory performance (bias corrected AU-ROC 0.757).

### Guideline compliance and MVPA

Previous studies show a large variation in PA guideline compliance, which partly reflect different guideline recommendations. Studies have shown the proportion of women who achieved 150 MVPA minutes/week based on total MVPA minutes dropped by approximately 50 % after extracting exclusively MVPA in bouts ≥10 min [42, 43]. Conceptually, the restriction of MVPA to bouts of activity corresponds better with studies based on self-reported PA, since questionnaire items typically refer to PA restricted to bouts [44]. Our findings are in accordance with studies of guideline compliance (≥150 MVPA minutes/week) based on self-reported PA which have shown that 11–32 % of pregnant women meet the target [15, 45, 46]. There are no population-based cohort studies that use objectively recorded MVPA restricted to bouts, but results from smaller studies of predominantly White healthy women suggest that 28–45 % of pregnant women comply with the PA guideline [42, 43]. The allowance of 2-min-interruptions within bouts and the homogeneous population are possible explanations why compliance was higher in those studies [42, 43]. We observed ethnic differences in prevalence of non-compliance and proportions with no recorded MVPA. Given that MVPA in bouts reflects recreational and transport activities better than MVPA without restriction, the ethnic difference may indicate that ethnic minority women perform such activities less frequently, or at intensities <3 METs. Previous population-based studies in Scandinavia have not addressed ethnic differences in PA in pregnancy [14, 47], but we found similar ethnic differences in MVPA, not restricted to bouts, from the current cohort in early pregnancy [19]. In agreement with our findings, a population based study from US using objectively recorded PA from pregnant women showed that non-Hispanic Black women recorded less MVPA than White women [16]. As the ethnic composition of our sample is different, our study contributes new and important evidence highlighting that ethnic differences in physical activity in mid-/late pregnancy is a public health concern in Northern Europe. Future research should explore mechanisms underlying these differences.

### Prediction of non-compliance with the PA guideline

The prognostic analysis presented is best described as a combined development and validation study [21], and as far as we are aware, it is the first report of a prognostic model development for PA guideline non-compliance in pregnancy. It was our motivation to extend the utility of predictors, from providing odds ratios (reflecting groups’ probabilities of non-compliance), to a model that could discriminate those who comply from those who do not comply with the PA guideline. The prognostic model of non-compliance with the PA guideline consisted of ethnicity, parity, physically active friends and body fat percentage.

The strong association observed between multiparity and non-compliance has been reported consistently [14, 47]. We found no significantly increased risk for uniparas (OR 1.2), probably due to few uniparas in the sample. While causal associations cannot be determined in the present study, our results concur with studies indicating special approaches are needed to promote PA among pregnant women with children.

To our knowledge, the observed positive association between many physically active friends and PA guideline compliance has not been reported previously in studies of pregnant women. A positive association between maternal PA and PA level of the spouse partly lends support to our finding [48]. In another Norwegian pregnancy cohort, no association was observed between exercise and the perceived exercise habits of friends [49]. The conflicting finding may partly reflect that exercise was self-reported and assessed at a later stage of pregnancy in a highly educated population [49]. Our finding suggests that a perception of having few physically active friends is a relevant predictor of PA guideline non-compliance in socially heterogeneous populations.

Our study showed that the probability of non-compliance with the PA guideline in GW 28 was strongly associated with body fat percentage in GW 15, and this finding concurs with previous reports of an inverse association between BMI and PA [14]. While BMI measures are more accessible in a primary health care setting, we decided to use bio-impedance derived body fat percentage based on reports of ethnic differences in the ratio between body fat and BMI [50]. Surprisingly, pre-pregnancy PA was not associated with PA guideline non-compliance in the final model. It seems plausible that the association between body fat percentage and non-compliance partly mediates the association between pre-pregnancy PA and PA level in pregnancy. Pre-pregnancy PA was self-reported and the lack of association may be explained by poor agreement between self-reported and objectively recorded PA [44, 51]. To our knowledge, an associations between pre-pregnancy PA and PA in pregnancy manifest only in studies based on self-reported PA at both time points [52, 53]. Hence, health care workers should be cautious in making inferences based upon self-reported PA as a measure of the true PA level.

The four predictors in the final prognostic model were strongly associated with PA guideline non-compliance, but this does not guarantee correct discrimination between women who did comply versus women who did not comply [54], and we observed only a fair discriminatory performance. Since measures of discriminatory performance may supplement odds ratios with information about the probability of non-compliance of an individual [55], we encourage integration of such measures in future studies to, hopefully, develop prognostic model with a better discriminatory performance.

### Strengths and weaknesses

The present study has several strengths such as the objectively recorded PA, the prospective design, the population-based sample, inclusion of a high proportion of ethnic minority women often excluded in research, a wide range of theoretically informed variables including psychosocial variables related to PA, and a high attendance rate [27]. Compared with other frequently used methods for objective PA recording (such as accelerometry), the SWA is considered more user-friendly and accurate [56]. Furthermore, we used bouts of MVPA ≥10 min, which is more strongly associated with health outcomes in the general populations, but is less studied in pregnancy. The ethnic composition of the cohort was representative for the largest ethnic groups of pregnant women in the participating city districts [27], probably making the study relevant to the pregnant populations in other European countries. While external validation of a prognostic model is optimal, it is often not feasible. Hence, we used the bootstrap procedure to correct for over-fitting, which is considered the optimal internal validation method [25].

However, this study also has weaknesses. In total, 33 % of the original cohort had incomplete or missing SWA data. A higher drop-out among ethnic minorities may have biased the estimates of MVPA minutes/week and PA guideline compliance. However, associations and the odds ratios are less prone to bias [57]. While energy expenditure recorded with other SWA models have been validated among pregnant women [29, 30], the model used in the present study has not been formally validated. However, estimates of energy expenditure does not differ significantly between the models [58]. Including SWA data from individuals with a minimum of 2 valid days in analyses deviates from the recommended minimum of 3–5 valid days [31]. However, by requiring wear time ≥19.2 h/day, an even lower number of valid days has been deemed sufficient [59]. Sensitivity analysis based on ≥4 valid days yielded similar odds ratios. Finally, wearing the SWA may have motivated participants to extend periods of MVPA.

## Conclusion

The low prevalence of PA guideline compliance (25 %) in GW 28 and the relatively large proportion (25 %) of women who never recorded MVPA in bouts ≥ 10 min are causes for concern from a public health perspective. Despite the higher prevalence of PA guideline non-compliance in certain risk groups, the overall non-compliance highlights the need for interventions reaching all pregnant women. The development of a prognostic model showed that the most important predictors of guideline non-compliance were ethnic minority background, multiparity, few physically active friends and high body fat percentage. While the odds ratios were highly significant, the model performed fairly well in discriminating between women who did comply and did not comply with the PA guideline. No previous studies of PA in pregnancy have included assessments of the discriminatory performance of predictors. To inform the risk assessments made by antenatal health care staff as part of their lifestyle counselling, future research should integrate measures of discriminatory performance in prospective studies of PA during pregnancy.

## Abbreviations

AU-ROC, area under the receiver operating characteristic; BMI, body mass index; GDM, gestational diabetes mellitus; GW, gestational week; MET, metabolic equivalents; MVPA, moderate-to-vigorous intensity physical activity; OR, odds ratio; PA, physical activity; SD, standard deviation; STORK-G, stork-groruddalen cohort study; SWA, Sensewear Armband

## Additional files

Additional file 1: Operationalization of variables. Detailed presentation of the operationalization of candidate predictors considered for analysis not included in the full model. (PDF 220 kb) Additional file 2: Predictor selection procedure. Presentation of the step-by-step procedure used for identifying candidate predictors and selecing the final predictors included in the prognostic model. (PDF 152 kb)
