# Supplementary material for: Predicting who fails to meet the physical activity guideline in pregnancy: a prospective study of objectively recorded physical activity in a population-based multi-ethnic cohort
Source: BMC Pregnancy Childbirth. 2016 Jul 26;16:186. doi: 10.1186/s12884-016-0985-x (PMC4962490; doi:10.1186/s12884-016-0985-x)
Supplement: Additional file 1: — Operationalization of variables. Detailed presentation of the operationalization of candidate predictors considered for analysis not included in the full model. (PDF 220 kb) [file 12884_2016_985_MOESM1_ESM.pdf]

Additional file 1: Operationalization of variables

- Candidate predictors with P<0.2 not included in the full model are presented below by domain
- Candidate predictors included in the full model are presented in the main article

Domain: Health

- **Health pre-pregnancy** was self-reported as “poor”, “not too good”, “good” or “very good” with the two former categories later merged.
- **Pelvic girdle-/lumbopelvic pain** was located by subjects to lumbar and/or anterior/posterior pelvis [1], and graded as “none”, “mild ”, “severe pain”. In analyses, pain was treated as a binary outcome (yes/no) and both single and multiple pelvic girdle pain sites were categorised as “yes”.
- **Annual weight change 18yr – current pre-pregnant weight** was calculated by dividing the weight difference by number of years since age 18.
- **Body mass index** (BMI, kg/m^2) pre-pregnancy was calculated from self-reported pre-pregnancy weight and body height measured at inclusion.

Domain: Culture

- **Residency period in Norway** was self-reported number of years since arrival in Norway. The continuous variable was categorized as “<2 to 4 years”, “1st generation and 2nd generation with 5-19 years in Norway”, “≥20 years or 2nd generation with uninterrupted residency in Norway”
- **Norwegian language skills** was self-reported as “very good”, “good”, “mediocre”, “somewhat poor”, and “poor”. In analyses we used three categories due to low cell counts. We merged “good” and “mediocre”, and, “somewhat poor” and “poor”, respectively.

Domain: Socioeconomic position

- **Educational level** referred to highest level of completed education. We categorized as “<10 years”, “10-12 years” or “university or college”.

Domain: Lifestyle

- **Smoking 3 months pre-pregnancy** was reported as “never”, “irregular”, and “daily”, with the two latter categories merged.

Domain: PA psychosocial

- **PA injuctive norms** was self-reported with reference to five items covering perceptions of important others’ opinion about the participant’s physical activity. The response categories for all items were “completely disagree=1”, “partly disagree=2”, “partly agree=3”, completely

agree=4”. Since questions 4 and 5 were negatively loaded, these were reversed. A sum score average based on all items was used in analyses. The questions were: Do important other:

1. think I should be physically active
2. think it is good for me to be physically active
3. want for me to be physically active
4. think it is inappropriate for me to be physically active
5. dislike that I am physically active.

- **PA self-efficacy** was reported with reference to five items using a 7-point Likert scale (1=”completely agree” and 7=”completely disagree”). Item 1, 2 and 4 were reversed to gain a positive correlation between self-efficacy and correlation. A sum score average based on all items was used in analyses. The statements were:
  1. Being physically active is completely my own choice
  2. If I wanted to, I would not find it difficult to do regular physical activity
  3. I would like to do regular physical activity, but I am not confident I would succeed
  4. I am in full control of being in regular physical activity
  5. To be in regular physical activity is hard for me.
- **PA identity** was reported with reference to three items using a 5-point Likert scale (1=”Not true” and 5= “True”). A sum score average based on all items was used in analyses. The item statements were:
  1. I see myself as a person who is conscious about being physically active
  2. I think om myself as a person who is conscious about being fit
  3. To be physically active is an important part of who I am.

Domain: Perceived preventive effect of PA

- Presented in the main article

Domain: Physical neighbourhood

- No predictors with $p<0.2$ in univariate logistic regression analysis. Therefore not presented

References

1. Albert H, Godskesen M, Westergaard J. Incidence of four syndromes of pregnancy-related pelvic joint pain. Spine (Philadelphia, Pa 1976). 2002;27:2831-2834
