# Supplementary material for: Predicting who fails to meet the physical activity guideline in pregnancy: a prospective study of objectively recorded physical activity in a population-based multi-ethnic cohort
Source: BMC Pregnancy Childbirth. 2016 Jul 26;16:186. doi: 10.1186/s12884-016-0985-x (PMC4962490; doi:10.1186/s12884-016-0985-x)
Supplement: Additional file 2: — Predictor selection procedure. Presentation of the step-by-step procedure used for identifying candidate predictors and selecing the final predictors included in the prognostic model. (PDF 152 kb) [file 12884_2016_985_MOESM2_ESM.pdf]

Additional file 2: Predictor selection procedure for the prognostic model

Step 1:

- Based on PA correlates identified in previous studies candidate predictors (Table, Column B) were selected and organized into domains (Table Column A).

Step 2:

- Univariate logistic regression was performed, and only predictors with p<0.2 were retained (Table, Column C):
- In each domain the predictor with lowest *P*-value was coined “preferred” while the other predictor(s) were coined “alternative” (Table, Column C). If the association in univariate regression was non-significant (p≥0.2), the cell inn column C was left empty.
- All preferred predictors were entered in multiple logistic regression to assess multivariable significance.
- To determine if an alternative predictor yielded a lower *P*-value, the preferred predictor was replaced by the alternative predictor in one domain, while the preferred predictors in the other domains were retained
- The procedure was repeated for alternative predictors in all domains.

Step 3:

- From each domain the candidate predictor with lowest *P*-value (as assessed by the repeated multiple logistic regression analyses in step 2) was entered in the full model. These were ethnicity, occupation, parity, body fat percentage, Self-reported PA, physically active friends and perceived preventive effect of PA.

Step 4:

- We performed multiple logistic regression with backward elimination to determine which predictors to include in the final prognostic model. Only variables with *P*-value ≤0.05 were retained in the final model. The following four predictors remained significant and were included in the final prognostic model: ethnicity, parity, body fat percentage, and physically active friends.

Step 5:

- As a sensitivity analysis, missing values for predictors included in the full model were imputed by chained equations to assess potential bias in complete case analyses. We analysed 20 replications [1]. The results of the full model yield similar results (data not shown).
- Details on missing values are presented in column D

Table

A	B	C	D
**Domains**	**Candidate predictors identified and available in data set**	**P<0.2 in univariate regression**	**Missing values**
Health	Body fat percentage visit 1	Preferred	No missing
	Health pre-pregnancy (Self-reported)	Alternative	5
	Pelvic girdle- /lumbopelvic pain	Alternative	9
	Annual weight change 18yr – current pre-pregnant	Alternative	16
	Age		No missing
	Recent Psychological trauma		18
	Lumbar pain (without pelvic pain)		9
Culture	Ethnicity	Preferred	No missing
	Residency period in Norway	Alternative	1
	Norwegian language skills	Alternative	No missing
	Use of interpreter		No missing
Socioeconomic position	Occupation	Preferred	7
	Education	Alternative	2
Pregnancy	Parity	Preferred	No missing
	Planned pregnancy	Alternative	5
	Pregnancy emesis		11
Lifestyle	Self-reported PA	Preferred	15
	Sleep duration		61
	Smoking 3 months pre-pregnancy		3
PA Psychosocial	Physically active friends (descriptive norm)	Preferred	20
	PA injunctive norm	Alternative	30
	PA self-efficacy	Alternative	14
	PA identity	Alternative	21
	PA perception of control		19
	PA family support		16
	PA peer support		13
	PA active neighbours		16
Perceived preventive effect of PA	Perceived preventive effect of PA	Preferred	19
Physical neighbourhood	Perception of physical activity opportunities		18
	Perception of walkability		18
	Perception of access to local shops		15
	Perception of access to local parks		19
	Perception of access to local exercise facilities		31

Reference

1 White IR, Royston P, Wood AM. Multiple imputation using chained equations: Issues and guidance for practice. *Stat Med* 2011;30:377-99.
